# Myostatin Orchestrates miR-124-Mediated Epigenetic Silencing of *ITGB1* to Suppress Skeletal Muscle Growth

**DOI:** 10.3390/biom16071012

**Published:** 2026-07-10

**Authors:** Qi Chen, Xianyu Wen, Mengting Li, Yingfang Niu, Wensai Yu, Yao Yao, Wei Wei, Jie Chen, Lifan Zhang

**Affiliations:** College of Animal Science and Technology, Nanjing Agricultural University, Nanjing 210095, China

**Keywords:** myostatin, miR-124, *ITGB1*, skeletal muscle

## Abstract

Although myostatin is a well-established inhibitor of myogenesis, its downstream mediators remain poorly characterized. This study identified integrin β1 (*ITGB1*), an integrin family member, as a novel mediator in myostatin signaling. Our results demonstrated that *ITGB1* promoted C2C12 myoblast differentiation. Myostatin treatment significantly downregulated *ITGB1* expression and suppressed myogenic differentiation, whereas *ITGB1* overexpression reversed the inhibitory effects of myostatin. Bioinformatic prediction and dual-luciferase reporter assay revealed that miR-124 suppressed *ITGB1* expression by targeting its 3′ untranslated region (UTR). Importantly, miR-124 reduced C2C12 myoblast differentiation by suppressing *ITGB1*. Furthermore, myostatin treatment markedly enhanced the expression of miR-124 in C2C12 myoblasts, thereby suppressing *ITGB1*. In vivo, blocking the myostatin/miR-124/*ITGB1* pathway significantly promoted skeletal muscle growth in mice. Accordingly, these data revealed that myostatin negatively regulated skeletal muscle growth in mice by promoting the miR-124-mediated epigenetic repression of *ITGB1*. Our findings provide new insights into the regulation of myogenesis via myostatin signaling.

## 1. Introduction

As a highly dynamic and metabolically active tissue constituting the majority of the body mass of vertebrates, skeletal muscle serves as a critical regulator in maintaining functional homeostasis of the body. In general, the developmental trajectory of skeletal muscle involves myoblast proliferation, differentiation, and subsequent fusion into muscle fibers [1], and this trajectory is regulated by many factors, including myogenic regulatory factors [2], myokines [3], and non-coding RNAs [4,5]. Pathological disruptions in skeletal muscle’s regenerative capacity or contractile functions frequently lead to myopathies that significantly compromise quality of life and longevity. Elucidating the molecular underpinnings of skeletal muscle growth may identify potential targets for the treatment of skeletal muscle disorders and age-related sarcopenia.

Myostatin is a powerful, negatively regulated myokine involved in skeletal muscle growth in humans and animals. Numerous studies have shown that myostatin inactivation causes skeletal muscle hypertrophy, whereas myostatin overexpression or systemic administration can lead to muscle atrophy [6,7,8]. Most recently, deletion of myostatin promoted myogenic commitment exhibited by muscle stem cells and muscle fiber cross-sectional area in mice [9,10]. Although the function of myostatin has been widely studied, the molecular mechanism underlying myogenesis inhibition has not been fully elucidated.

Integrins, heterodimeric transmembrane receptors composed of α and β subunits, serve as a crucial link between extracellular matrix components and intracellular cytoskeleton networks [11]. Integrin β1 (*ITGB1*), a member of the most prominent integrin subfamily, is essential for cell adhesion and migration [12]. *ITGB1* affects multiple stages of muscle generation, including myoblast migration, differentiation, fusion into muscle ducts, and formation of sarcomeres [13,14]. Transcriptomic data of C2C12 cells treated with myostatin also identified *ITGB1* as a possible target crucial for the inhibitory effect of myostatin on myoblast differentiation [15]; however, the mechanism is currently unclear as to how myostatin function is achieved through *ITGB1*. To shed light on this issue, this study systematically deciphered the functional role and molecular mechanism of *ITGB1* in myostatin-mediated inhibition of myoblast differentiation.

## 2. Materials and Methods

### 2.1. Animals

C57BL/6JGpt mice (Strain NO. N000013, Mouse Genome Informatics (MGI), http://www.informatics.jax.org/) were purchased from GemPharmatech (Nanjing, China). CRISPR-Cas9-mediated gene editing was employed to produce knockout (KO) mice, targeting a 215 bp segment of the miR-124 promoter containing the SMAD family member 4 (SMAD4)-binding site (AGAC), which was discovered in our previous study to be necessary for myostatin activation of miR-124 [16]. Briefly, using the AGAC sequence within the promoter region of the mouse miR-124 gene (Gene ID: 723951) as a target for knockout, potential gRNA sequences were predicted by inputting the flanking sequences of the target site. Off-target analysis was performed based on the GRCm38/mm10 genome assembly. Among all candidate gRNAs, two were selected based on their optimal positioning, minimal predicted off-target effects, and highest efficacy scores, with their sequences provided in Supplementary Table S1. Cas9 mRNA and gRNAs generated by in vitro transcription were co-injected into fertilized eggs for KO mouse production. Microinjected fertilized eggs were subsequently transferred into the oviducts of surrogate mice. After birth, the offspring were subjected to polymerase chain reaction (PCR) and sequencing to confirm the presence of positive founders. Specific PCR primers for the miR-124 promoter region, including this binding site, were designed to verify successful genomic modification and are detailed in Supplementary Table S1. Founder mice were randomly mated to produce offspring and their genotypes were confirmed by PCR (Supplementary Figure S1). All mice were housed in the Laboratory Animal Center of Nanjing Agricultural University, Nanjing, China, under optimal conditions of temperature, humidity, and light cycle. The mice had unfettered access to food. Three-month-old male mice were randomly stratified into four groups: PBS-injected wild-type (WT), PBS-injected KO, myostatin-injected WT, and myostatin-injected KO, with three mice per group. Myostatin was dissolved in PBS to prepare a 5 μg/mL concentrated stock solution, which was stored at −80 °C. Prior to each injection, the stock solution was diluted with phosphate-buffered saline (PBS) to a final dose of 200 ng/mL. The mice received bilateral intramuscular injections into the vastus lateralis once every two days for one month at a dosage of 2 ng/g body weight, with half the total volume administered to each side. Following anesthesia with 1.25% tribromoethanol at a dosage of 0.2 mL per 10 g body weight, mice were euthanized by cervical dislocation. All animal experiment procedures were approved by the Animal Ethics Committee of Nanjing Agricultural University, Nanjing, China (Approval No.20220318053).

The collected tissues from the bilateral vastus lateralis muscles were fixed in 4% paraformaldehyde and embedded in paraffin, followed by tissue slicing and staining with hematoxylin and eosin (HE staining). The cross-sectional area of the muscle tube was evaluated using Image pro plus software.

### 2.2. Cell Culture and Treatment

The C2C12 (ZQ0092) (CVCL_0188 in the Cellosaurus database, www.cellosaurus.org) and 293T (ZQ0033) (CVCL_0063 in the Cellosaurus database, www.cellosaurus.org) cell lines were purchased from Zhong Qiao Xin Zhou Biotechnology Co., Ltd. (Shanghai, China). Cells were cultured in growth medium (GM) consisting of high-glucose Dulbecco’s modified Eagle’s medium (DMEM) supplemented with 10% fetal bovine serum and 1% penicillin–streptomycin. C2C12 cells were cultured in differentiation medium (DM) containing high-glucose DMEM supplemented with 2% horse serum and 1% penicillin–streptomycin for 5 days, with the medium changed every 2 days during this period. Myostatin recombinant protein (Ze Ye, Shanghai, China) was diluted in PBS to achieve a working concentration of 5 μg/mL. C2C12 cells were treated with various concentrations of myostatin for 24 h and then harvested to determine the effect of myostatin on the expression of *ITGB1*. C2C12 cells were then treated with 0.25 μg/mL myostatin to detect its effect on myoblast differentiation.

### 2.3. Plasmid Construction, RNA Oligonucleotides, and Cell Transfection

The PCR product of *ITGB1* coding sequence (CDS) was cloned into the pcDNA3.1 vector, using EcoRI and XhoI restriction endonucleases. The pmirGLO-ITGB1-3′UTR wild type (ITGB1-wt) dual-luciferase miRNA target expression vector was synthesized by Qinke (catalog number: NJ0065293) (Beijing, China). The pmirGLO-ITGB1-3′UTR mutant type (ITGB1-mut) dual-luciferase vector was constructed by replacing the predicted target sequence bases of miR-124 in the wild vector using a Mut Express II fast mutagenesis kit (Vazyme Biotech, Nanjing, China). All RNA oligonucleotides, including miR-124 mimics, mimics negative control (mimics NC), miR-124 inhibitor, and inhibitor NC, were purchased from GenePharma (Shanghai, China). The pcDNA3.1(+) vector, mimics NC, and inhibitor NC were used as experimental controls. *ITGB1* overexpression plasmid vector, miR-124 mimics, miR-124 inhibitor, or controls were introduced into cells using a Lipofectamine 3000 kit (Invitrogen, Carlsbad, CA, USA). Sequences of the above oligonucleotides are listed in Supplementary Table S1. The wild-type promoter sequence (pGL4-WT) and *SMAD4* binding site-mutated promoter sequence (pGL4-MUT) of miR-124 (Supplementary Table S2) were individually cloned into the pGL4-basic vector (catalog number: C09005 for pGL4-WT and C09006 for pGL4-MUT). The CDS of *SMAD4* was inserted into pcDNA 3.1(+) to construct a pcDNA-*SMAD4* overexpression vector (catalog number: C05008), with the pGL4-RL vector serving as the internal control. All vectors were synthesized and purified by Suzhou Genepharma Co., Ltd., Suzhou, China.

### 2.4. Target Gene Prediction

Target genes were predicted using three bioinformatics tools, including miRTarBase (https://awi.cuhk.edu.cn/miRTarBase), TargetScan (http://www.targetscan.org/mmu_72/) (accessed on 27 March 2022). The common genes predicted by at least two databases were designated as potential target genes of miR-124.

### 2.5. Dual-Luciferase Reporter Assays

HEK293T cells in logarithmic growth phase were digested and seeded into 24-well plates at a density of 1 × 10^5^ cells/well, followed by incubation at 37 °C with 5% CO_2_ for 24 h. When cell density reached 70–80%, two sets of co-transfections were performed using the GP-transfect-Mate transfection reagent (Genepharma, Suzhou, China) following the manufacturer’s protocol. In the first set, 1 μg of *ITGB1*-wt or *ITGB1*-mut was co-transfected with 50 nM miR-124 mimics or NC; in the second set, pGL4-basic, pGL4-WT, or pGL4-MUT was co-transfected with pcDNA3.1 or pcDNA-*SMAD4*. At 24 or 48 h post-transfection, cell samples were prepared for luciferase activity measurements using a dual-luciferase reporter assay system (Promega, Madison, WI, USA). Luminescence intensities were measured using a multimode microplate reader (PerkinElmer EnSpire, Shelton, CT, USA), and the ratio of firefly to Renilla luciferase activity was calculated.

### 2.6. Quantitative Real-Time PCR (qRT-PCR)

Total RNA was isolated from tissues or cells using TRIzol reagent (ABclonal, Wuhan, China). Reverse transcription was performed using an Evo M-MLV RT Kit (Accurate Biotechnology, Hunan, China) for gene transcripts, and the miRNA 1st Strand cDNA Synthesis Kit (Vazyme Biotech, Nanjing, China) for miRNA-124 and U6. For the latter, the stem-loop primer for miRNA-124 and the reverse primer for U6 were included in the reaction system. Genious 2X SYBR Green Fast qPCR Mix (ABclonal, Wuhan, China) was used for qRT-PCR analysis. miRNA Universal SYBR qPCR Master Mix (Vazyme Biotech, Nanjing, China) was used to determine the expression of miR-124. *RPLP0* and *U6* were selected as the endogenous references for these genes and miR-124, respectively. Finally, the relative levels of genes and miR-124 were determined by the 2^−ΔΔCt^ method. All primers used in qRT-PCR are listed in Supplementary Table S1.

### 2.7. Immunofluorescence Analysis

After 24 h transfection, C2C12 cells were induced to differentiate for 5 days. Subsequently, the differentiated cells were washed three times with PBS and fixed with 4% paraformaldehyde for 30 min. The permeabilized cells were treated with 0.3% Triton X-100 for 30 min and incubated with 5% bovine serum albumin for 2 h. Primary antibody of myosin heavy chain (MYHC, catalog number: A25357; clone ID: ARC62607, Abclonal, Wuhan, China) incubation was conducted overnight at 4 °C, with subsequent incubation of the secondary antibody (CY3-labeled IgG, catalog number: A0516, Beyotime Biotechnology, Shanghai, China) at a normal indoor temperature for 2 h. DAPI (Biosharp, Beijing, China) was used to stain the nucleus under light-free conditions. Fluorescence imaging was captured using a fluorescence microscope (Zeiss, Oberkochen, Germany) and evaluated using ImageJ software (version 1.53k). The fusion index (FI) was utilized for evaluating myotube formation, with its value computed using the formula FI = (Number of Nuclei In/Total Number of detected Nuclei) × 100.

### 2.8. Western Blot

Total protein extracts from tissues or cells were obtained and quantified using RIPA extraction reagent and a bicinchoninic acid (BCA) protein assay kit (Beyotime Biotechnology, Jiangsu, China), respectively. Equivalent amounts of protein were electrophoresed on 12% sodium dodecyl sulfate-polyacrylamide gel electrophoresis gels and transferred onto PVDF membranes. The membranes were blocked with 5% BSA and then probed with specific primary antibodies, including MyHC (catalog number: A25357; clone ID: ARC62607), myogenin (MyoG, catalog number: A24206; clone ID: ARC65610), ITGB1 (catalog number: A27421), and β-Actin (1:1000, Abclona, Wuhan, China) (catalog number: AC026; clone ID: ARC5115-01) overnight at 4 °C. The membranes were then incubated with an anti-rabbit/goat IgG secondary antibody (catalog number: A0208) at room temperature for 2 h. Protein bands were visualized using a Tanon 5200 instrument (Tannon, Shanghai, China) and quantified using ImageJ software (version 1.53k).

### 2.9. Statistical Analysis

Statistical comparisons were analyzed by two-tailed student’s *t*-test (two groups) or analysis of variance (ANOVA) (≥3 groups) with Tukey’s post hoc test using GraphPad Prism v.6 software (La Jolla, CA, USA). The results were presented in the form of mean ± standard error (SEM). A threshold of *p*-value less than 0.05 was adopted to determine statistical significance.

## 3. Results

### 3.1. ITGB1 Promoted Differentiation of C2C12 Myoblasts

**Figure 1:**
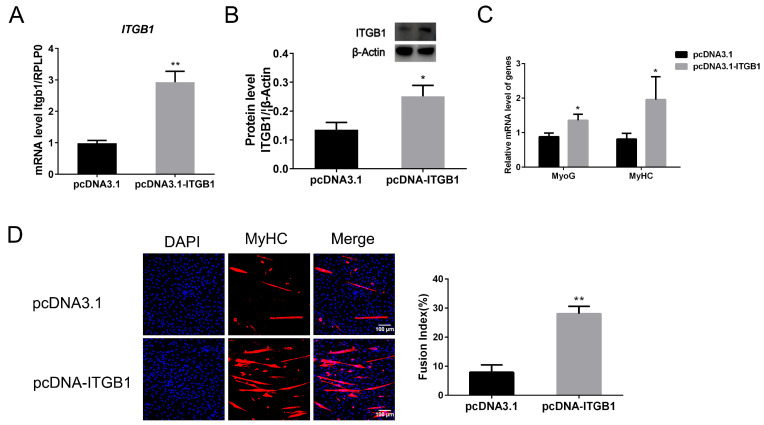
ITGB1 promotes differentiation of C2C12 myoblasts. (**A**) mRNA expression of ITGB1 in C2C12 cells transfected with pcDNA3.1 or pcDNA3.1-ITGB1 for 24 h. (**B**) Protein expression of ITGB1 in C2C12 cells transfected with pcDNA3.1 or pcDNA3.1-ITGB1 for 48 h. (**C**) mRNA expression of MyoG and MyHC on day 5 of myogenic differentiation in C2C12 cells transfected with pcDNA3.1 or pcDNA3.1-ITGB1. (**D**) Immunofluorescence analysis of myotube formation on day 5 of myogenic differentiation in C2C12 cells transfected with pcDNA3.1 or pcDNA3.1-ITGB1. Blue: nuclear staining; Red: MyHC staining; Scale bar = 100 μm. The data were presented as the mean ± SEM from at least three independent experiments. Results in panel (**A**–**D**) were analyzed using students’ *t*-test. * *p* < 0.05, ** *p* < 0.01. The original WB images are shown in Figure S2A.

Compared to cells transfected with pcDNA3.1(+), the mRNA and protein levels of *ITGB1* were significantly increased in cells transfected with pcDNA3.1-ITGB1 (*p* < 0.05 or *p* < 0.01) (Figure 1A,B), indicating that overexpression vector of *ITGB1* was successfully constructed. On day 5 of myogenic differentiation, *ITGB1* overexpression significantly upregulated the mRNA levels of myogenin (*MyoG*) and myosin heavy chain (*MyHC*) (*p* < 0.05) (Figure 1C) and significantly increased the formation of myotubes (*p* < 0.01) (Figure 1D).

### 3.2. Myostatin Regulated ITGB1 to Suppress C2C12 Myoblast Differentiation

**Figure 2:**
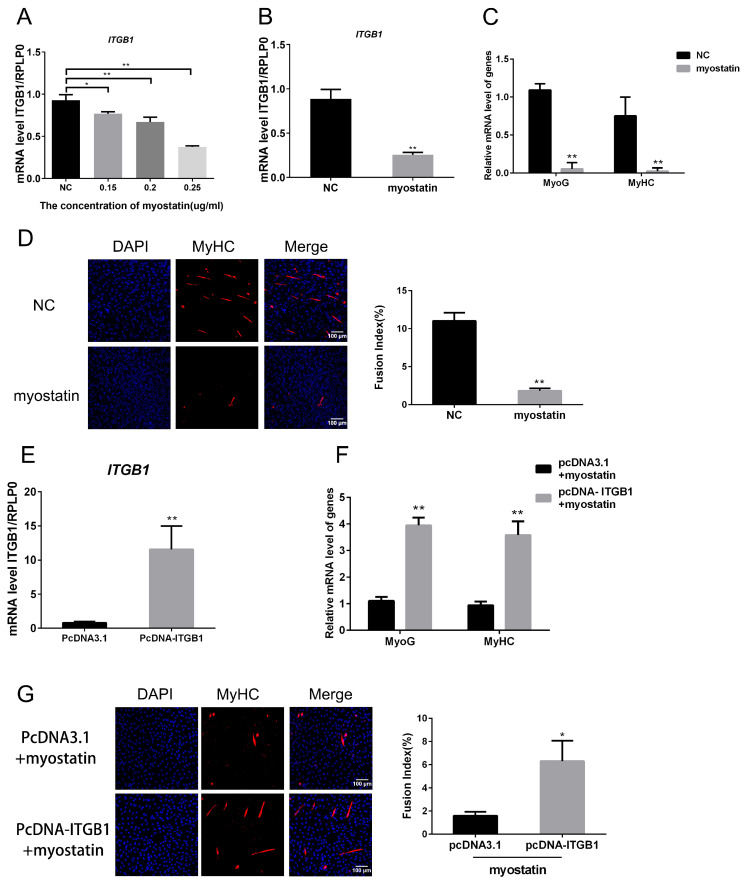
Myostatin regulates ITGB1 expression to suppress C2C12 myoblast differentiation. (**A**) mRNA levels of ITGB1 in C2C12 cells treated with different myostatin concentrations for 24 h. (**B**,**C**) mRNA expression of ITGB1 (**B**), MyoG and MyHC (**C**) on day 5 of myogenic differentiation in C2C12 cells treated with myostatin. (**D**) Immunofluorescence analysis of myotube formation on day 5 of myogenic differentiation in myostatin-treated C2C12 cells. (**E**,**F**) mRNA expression levels of ITGB1 (**E**), MyoG and MyHC (**F**) on day 5 of myogenic differentiation in C2C12 cells treated with myostatin or myostatin in combined with pcDNA3.1-ITGB1. (**G**) Immunofluorescence analysis of myotube formation on day 5 of myogenic differentiation in C2C12 cells treated with myostatin or myostatin in combination with pcDNA3.1-ITGB1. Blue: nuclear staining; Red: MyHC staining; Scale bar = 100 μm. The data were presented as the mean ± SEM from at least three independent experiments. Results in panel (**A**–**G**) were analyzed using students’ *t*-test. * *p* < 0.05, ** *p* < 0.01.

Treatment with different concentrations of myostatin significantly downregulated the mRNA expression of *ITGB1* (*p* < 0.05 or *p* < 0.01) (Figure 2A). Subsequently, 0.25 μg/mL myostatin was selected as the treatment concentration for the further experiments. mRNA expression levels of *ITGB1*, *MyoG*, and *MyHC* were significantly downregulated in differentiated cells treated with myostatin (*p* < 0.01) (Figure 2B,C). Moreover, immunofluorescence analysis showed that myostatin treatment reduced myotube formation in differentiated cells (*p* < 0.01) (Figure 2D). Compared to cells treated with myostatin alone, mRNA levels of *ITGB1*, *MyoG*, and *MyHC*, as well as myotube formation, were significantly increased in cells co-treated with myostatin and pcDNA3.1-ITGB1 (*p* < 0.05 or *p* < 0.01) (Figure 2E–G). These data indicated that *ITGB1* overexpression rescued the inhibitory effect of myostatin on myoblast differentiation.

### 3.3. MiR-124 Suppressed C2C12 Myoblast Differentiation by Regulating ITGB1

**Figure 3:**
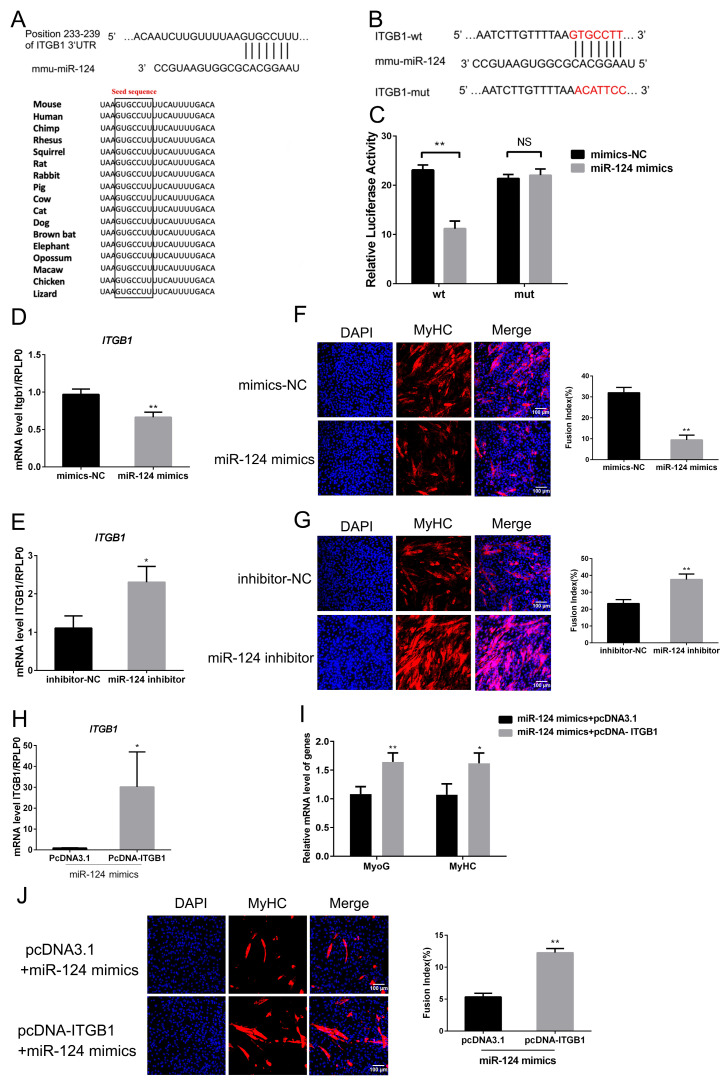
MiR-124 suppresses myoblast differentiation by targeting ITGB1. (**A**) Sequence of miR-124 and its conservative binding region in the 3′UTR of ITGB1. (**B**) Sequence comparisons of wild-type and mutant fluorescent vectors based on the binding site of miR-124 and the 3′UTR of ITGB1. (**C**) Relative luciferase activity of 293T cells transfected with miR-124 mimics or NC for 24 h. (**D**,**E**) The mRNA expression of ITGB1 after transfection with miR-124 mimics (**D**) or inhibitor (**E**). (**F**,**G**) Immunofluorescence analysis of myotube formation on day 5 of myogenic differentiation in C2C12 cells transfected with the miR-124 mimics (**F**) or inhibitor (**G**). (**H**,**I**) mRNA levels of ITGB1 (**H**), MyoG, and MyHC (**I**) on day 5 of myogenic differentiation in cells transfected with miR-124 mimics or co-transfected with miR-124 mimics and pcDNA-ITGB1. (**J**) Immunofluorescence analysis of myotube formation on day 5 of myogenic differentiation in C2C12 cells transfected with miR-124 mimics or co-transfected with miR-124 mimics and pcDNA-ITGB1. Blue: nuclear staining; Red: MyHC staining; Scale bar = 100 μm. The data were presented as the mean ± SEM from at least three independent experiments. Results in panel (**C**–**J**) were analyzed using students’ *t*-test. NS: *p* > 0.05, * *p* < 0.05, ** *p* < 0.01.

Bioinformatics prediction identified 154 miR-124 target genes shared by at least two prediction tools. Comparative analysis of these genes and the existing transcriptomic data of myostatin-treated C2C12 cells [15] revealed that only *ITGB1* was shared between the two datasets, indicating that *ITGB1* serves as the primary functional mediator of the myostatin/miR-124 axis. Meanwhile, miR-124 is capable of targeting the 3′UTR of *ITGB1* and the mature sequence of miR-124 is highly conserved among vertebrates (Figure 3A). We constructed ITGB1-wt and ITGB1-mut plasmids (Figure 3B). The dual-luciferase reporter assay revealed that overexpression of miR-124 significantly suppressed cell activity in the ITGB1-wt group (*p* < 0.01), but had no significant effect in the ITGB1-mut group (*p* > 0.05) (Figure 3C). After transfection with miR-124 mimics and inhibitor, the mRNA expression of *ITGB1* was significantly decreased and increased, respectively (*p* < 0.05 or *p* < 0.01) (Figure 3D,E), indicating that miR-124 could specifically target *ITGB1* 3′UTR to regulate *ITGB1* expression. Immunofluorescence analysis revealed a significant decrease and increase in myotube formation after transfection with miR-124 mimics and inhibitor, respectively (*p* < 0.01) (Figure 3F,G), implying that miR-124 is a negative regulator of myoblast differentiation.

Compared with the group transfected with miR-124 mimics alone, the mRNA levels of *ITGB1*, *MyoG*, and *MyHC* were significantly upregulated after co-transfection with miR-124 mimics and pcDNA-ITGB1 (*p* < 0.05 or *p* < 0.01) (Figure 3H,I). Immunofluorescence analysis also revealed a significant increase in myotube formation in the co-transfection group (*p* < 0.01) (Figure 3J). Notably, overexpression of *ITGB1* rescued the inhibitory effects of miR-124 mimics on myoblast differentiation.

### 3.4. Myostatin Suppressed ITGB1 Expression Through Upregulating miR-124

**Figure 4:**
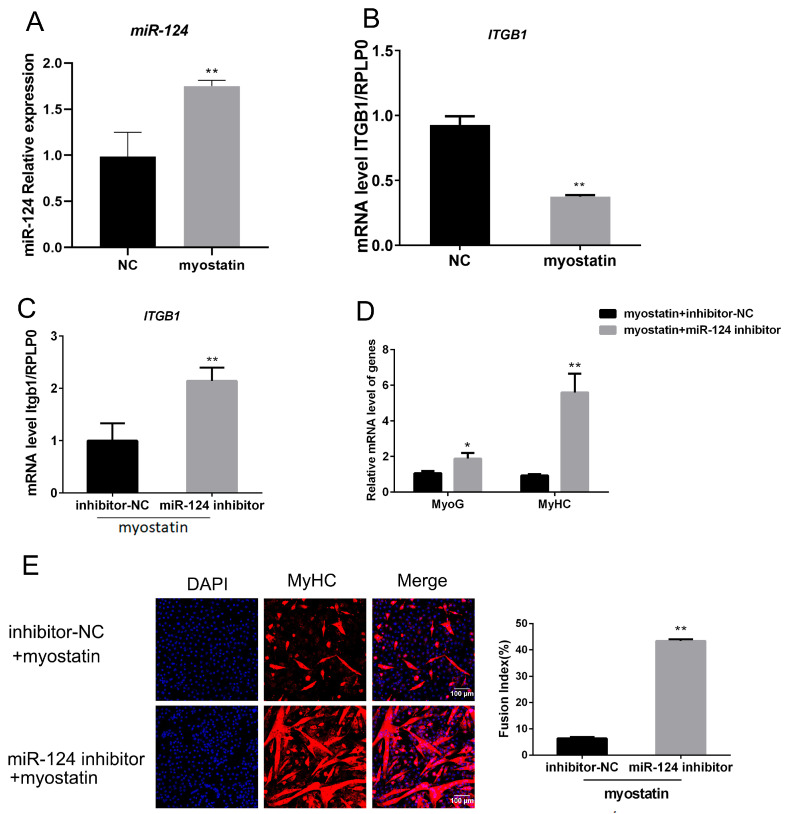
Myostatin suppresses ITGB1 expression by upregulating miR-124 expression. (**A**) mRNA expression of miR-124 in C2C12 cells treated with myostatin for 24 h. (**B**) mRNA expression of ITGB1 in C2C12 cells treated with myostatin for 24 h. (**C**,**D**) mRNA levels of ITGB1 (**C**), MyoG, and MyHC (**D**) on day 5 of myogenic differentiation in C2C12 cells treated with myostatin combined with inhibitor NC or miR-124 inhibitor. (**E**) Immunofluorescence of myotube formation on day 5 of myogenic differentiation in C2C12 cells treated with myostatin combined with inhibitor NC or miR-124 inhibitor. Blue: nuclear staining; Red: MyHC staining; Scale bar = 100 μm. The data were presented as the mean ± SEM from at least three independent experiments. Results in panel (**A**–**E**) were analyzed using students’ *t*-test. * *p* < 0.05, ** *p* < 0.01.

Our previous study found that myostatin upregulates the levels of miR-124 in 3T3-L1 preadipocytes [16], and the effect of myostatin on miR-124 expression in myoblasts was further investigated. The results demonstrated that expressions of miR-124 and *ITGB1* were significantly upregulated and downregulated (*p* < 0.01) in myostatin-treated C2C12 cells, respectively (Figure 4A,B). Compared with myostatin-treated cells, the mRNA levels of *ITGB1*, *MoyG*, and *MyHC* were significantly upregulated in C2C12 cells treated with myostatin combined with the miR-124 inhibitor (*p* < 0.05 or *p* < 0.01) (Figure 4C,D). Immunofluorescence analysis demonstrated that miR-124 inhibitor rescued the inhibitory effect of myostatin on myotube formation in C2C12 cells (Figure 4E). These results confirmed that miR-124-mediated myostatin regulates *ITGB1* to suppress myoblast differentiation.

### 3.5. Blocking Myostatin/miR-124/ITGB1 Pathway Promoted Skeletal Muscle Growth in Mice

**Figure 5:**
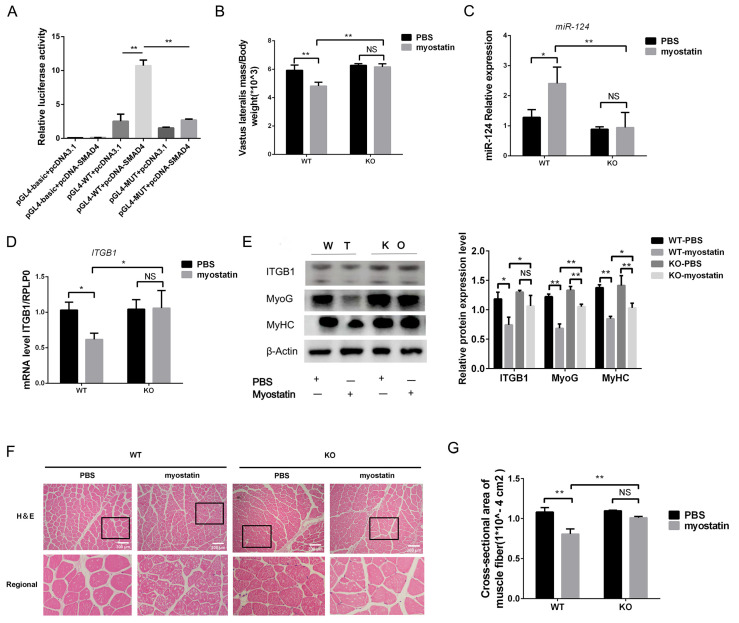
Blocking the myostatin/miR-124/ITGB1 pathway promoted skeletal muscle growth in mice. (**A**) Relative luciferase activity of miR-124 promotor region. pGL4-basic + pcDNA3.1: pGL4-basic reporter vector + pcDNA3.1 empty vector control; pGL4-basic + pcDNA-SMAD4: pGL4-basic reporter vector + SMAD4 overexpression vector; pGL4-WT + pcDNA3.1: Wild-type promoter + pcDNA3.1 empty vector control; pGL4-WT + pcDNA-SMAD4: Wild-type promoter + SMAD4 overexpression vector; pGL4-MUT + pcDNA3.1: Mutated promoter + pcDNA3.1 empty vector control; pGL4-MUT + pcDNA-SMAD4: Mutated promoter + SMAD4 overexpression vector. (**B**) The Ratio of vastus lateralis to body weight after 30 days of injection of PBS or myostatin into wild-type (WT) and knockout (KO) mice. (**C**,**D**) mRNA expression levels of miR-124 (**C**) and ITGB1 (**D**) in the vastus lateralis of WT and KO mice treated with PBS or myostatin. (**E**) Protein expression of ITGB1, MyoG, and MyHC in the vastus lateralis of WT and KO mice treated with PBS or myostatin. (**F**) Paraffin sections of the vastus lateralis of WT and KO mice treated with PBS or myostatin after HE staining. Scale bar = 300 μm. (**G**) Mean cross-sectional area of myotubes in paraffin-embedded sections from the vastus lateralis of WT and KO mice treated with PBS or myostatin. The data were presented as the mean ± SEM *(n* = 3 per group). Results in panel (**A**–**E**,**G**) were analyzed using one-way ANOVA followed by Tukey’s post hoc test. NS: *p* > 0.05, * *p* < 0.05, ** *p* < 0.01. The original WB images are shown in Figure S2B.

Our previous study showed that myostatin regulated miR-124 through activation of SMAD4, which combines with the promoter region of miR-124 to promote its expression in 3T3-L1 cells [16]. Here, our results demonstrated that luciferase activity in the pGL4-WT + pcDNA3.1 group was extremely significantly lower than that in the pGL4-WT + pcDNA-*SMAD4* group (*p* < 0.01), while luciferase activity in the pGL4-WT + pcDNA-*SMAD4* group was extremely significantly higher than that in the pGL4-MUT + pcDNA-*SMAD4* group (*p* < 0.01) (Figure 5A), demonstrating that mutation of the *SMAD4* binding site (from AGAC to AACT) within the miR-124 promoter region impaired the interaction between *SMAD4* and the miR-124 promoter, leading to a marked reduction in promoter transcriptional activity. These results confirmed that *SMAD4* regulates miR-124 transcription by directly binding to its promoter region. To obtain a myostatin/miR-124/*ITGB1* pathway-specific blocking model, we knocked out the miR-124 promoter region, including the SMAD4-binding site, in mice using the CRISPR-Cas9 system. Wild-type mice injected with myostatin had a significantly lower ratio of the vastus lateralis to body weight than wild-type mice injected with PBS (*p* < 0.01) (Figure 5B), whereas there were no significant changes in the ratio of vastus lateralis to body weight between knockout mice injected with myostatin and PBS (*p* > 0.05) (Figure 5B). Compared with myostatin-injected wild-type mice, the ratio of vastus lateralis to body weight was also significantly higher in myostatin-injected knockout mice (*p* < 0.01) (Figure 5B). Moreover, myostatin injection significantly upregulated and downregulated the expression of miR-124 and *ITGB1* in the vastus lateralis of wild-type mice, respectively (*p* < 0.05) (Figure 5C–E), but had no significant effect on the expression of miR-124 and *ITGB1* in the vastus lateralis of knockout mice (*p* > 0.05) (Figure 5C–E). The mRNA and protein levels of *ITGB1* of myostatin-injected knockout mice were significantly higher than those of myostatin-injected wild-type mice (*p* < 0.05) (Figure 5D,E). In both wild-type and knockout mice, protein levels of MyoG and MyHC were significantly downregulated after myostatin injection compared with those in the control group (*p* < 0.01) (Figure 5E). However, protein levels of MyoG and MyHC in the vastus lateralis of knockout mice injected with myostatin were significantly higher than those in wild-type mice injected with myostatin (*p* < 0.05 or *p* < 0.01) (Figure 5E). Compared to the control group injected with PBS, myostatin treatment significantly reduced the mean cross-sectional area of myotubes in the lateral femoris muscle of wild-type mice (*p* < 0.01) (Figure 5F,G). The mean cross-sectional area of myostatin-injected knockout mice remained significantly larger than that of myostatin-injected wild-type mice (*p* < 0.01) (Figure 5F,G). These data indicate that blocking the myostatin/miR-124/ITGB1 pathway promoted skeletal muscle growth in mice in vivo.

## 4. Discussion

As a pivotal member of the extensive transforming growth factor family, myostatin is mainly secreted in skeletal muscle and exhibits conserved functional characteristics across mammalian species. Myostatin exerts a negative regulatory influence on growth and morphogenesis of skeletal muscle by suppressing proliferation and differentiation of myoblasts and reducing skeletal muscle protein synthesis [17,18]. This negative regulator of skeletal muscle growth operates through complex regulatory processes involving the activin type II receptor and activin receptor-like kinase type I receptor complex formation, SMAD2/3 phosphorylation and SMAD4 binding, and SMAD2/3/4 transcriptional complex modulation of target gene expression [19]. A previous transcriptome study demonstrated that exogenous myostatin treatment downregulated the expression of hundreds of genes in differentiated C2C12 cells, including genes related to muscle development, such as *MEF2* (myocyte enhancer factor), *HGF* (hepatocyte growth factor), *ILB* (interleukin 1 beta), and *ITGB1* (integrin beta 1) [15], suggesting that *ITGB1* is a potential target gene regulated by myostatin.

Generally speaking, the interplay between myoblasts and extracellular matrix plays a key role in skeletal muscle growth. One mechanism by which the extracellular matrix relays signals into cells is through integrin receptors, which provide transmembrane linkages between the extracellular matrix and the cytoplasmic layer. Functioning as a pleiotropic constituent within the integrin family, *ITGB1* exerts an essential regulatory function in myogenesis [20] and is necessary for maintaining microcirculatory homeostasis in satellite cells and muscle tissue [21]. For example, TMEM182 impedes myoblast differentiation and fusion by binding to *ITGB1* [22], whereas *ITGB1* influences the migration and differentiation of bovine muscle satellite cells by interacting with secreted protein acidic and rich in cysteine (SPARC)-like 1 (SPARCL1) in the extracellular matrix [23]. Our study directly demonstrates that *ITGB1* overexpression in C2C12 myoblasts upregulates the key myogenic markers *MyoG* and *MyHC*. This finding confirms that *ITGB1* is a critical regulator of the C2C12 myoblast differentiation. Additionally, as myostatin functions as a negative regulator of myoblast proliferation via G1/S cell-cycle blockade and satellite-cell quiescence [24,25], ITGB1 conversely acts as a positive regulator. By synergizing with FGF2, ITGB1 activates ERK/Akt signaling to promote satellite cell proliferation and self-renewal [26], implying its potential involvement in the myostatin-mediated regulation of myoblast proliferation. However, the present study addresses only the differentiation axis, leaving this proliferative function unexamined and necessitating further systematic evaluation.

**Figure 6:**
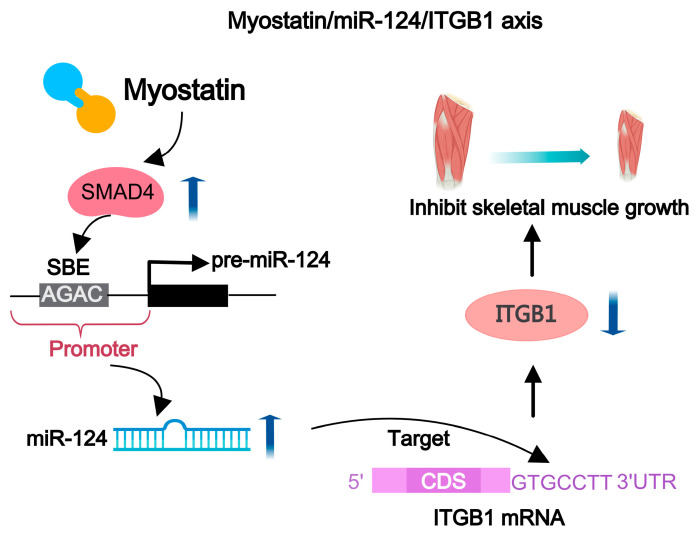
Schematic diagram of myostatin/miR-124/ITGB1 axis to regulate skeletal muscle growth. Created with MedPeer (medpeer.cn) (ID:fef66bazp66ji89mq1783677233).

Furthermore, miR-124 has been identified as a critical molecular link between myostatin signaling and *ITGB1*. Recently, several miRNAs, including miR-495 and miR-455-3p, were shown to play a mediating role in the process through which myostatin regulates downstream pathway genes, thereby facilitating the impact of myostatin on myogenesis [27,28]. In muscle biology, miR-124 has been shown to regulate the proliferation and autophagy of goat myoblasts by targeting and suppressing *VMP1* expression [29]. Moreover, it promotes the myogenic differentiation of adipose-derived stem cells through targeted inhibition of *CAV1*, thereby influencing myogenesis [30]. In contrast, we demonstrated that miR-124 suppressed myoblast differentiation, whereas overexpression of *ITGB1* rescued the inhibitory effect of miR-124 on myoblast differentiation, suggesting that the negative regulatory effect of miR-124 on muscle development in mice may be primarily achieved via integrin signaling. Additionally, our previous study showed that myostatin activated miR-124 by upregulating the expression of SMAD4, which binds to the miR-124 promoter region, thereby inhibiting the differentiation of 3T3-L1 preadipocytes [16]. Here, we demonstrated that myostatin plays a similar negative role in myoblast differentiation by regulating the expression of miR-124, suggesting that myostatin functions through the miR-124/*ITGB1* axis in vitro (Figure 6). More importantly, in a knockout mouse model in which the miR-124 promoter region containing the SMAD4 binding site was knocked out, we observed that when miR-124 expression was no longer regulated by SMAD4, the inhibitory effect of myostatin on skeletal muscle growth was partially abrogated. This further confirmed the existence of the myostatin/miR-124/*ITGB1* signaling pathway in vivo. Taken together, these data delineate a novel signaling pathway governing myoblast development that is regulated by myostatin signaling.

In addition, several limitations of this study should be acknowledged. First, our experimental design only explored the expression and functional regulation of myostatin in local murine skeletal muscle tissues, without quantifying its secretion or dynamic concentrations in the peripheral blood. This precludes a comprehensive evaluation of the systemic regulatory functions of myostatin mediated by humoral circulation. Second, while it is well established that miR-124 is sorted into exosomes for extracellular release and subsequent systemic transport, thereby enabling communication between distant cells and tissues [31], the present study confined its detection to the intracellular fraction of muscle cells. Consequently, the lack of exosomal miR-124 quantification in cell culture supernatants or peripheral biofluids precludes a comprehensive evaluation of its potential paracrine or endocrine functions. These considerations warrant further investigation.

## 5. Conclusions

This study established the myostatin/miR-124/*ITGB1* signaling axis as a novel regulatory circuit governing skeletal muscle growth. Mechanistically, we demonstrated that myostatin suppresses myogenic differentiation in mice both in vitro and in vivo through miR-124-mediated epigenetic repression of *ITGB1*. These findings not only elucidated the molecular cascade underlying the inhibitory effects of myostatin on myogenesis, but also suggest that miR-124 is a novel therapeutic strategy for combating skeletal muscle disorders.

## Data Availability

Data are available upon reasonable request.

## Supplementary Materials

The following supporting information can be downloaded at: https://www.mdpi.com/article/10.3390/biom16071012/s1, Table S1. Information of primers or oligonucleotides used in the present study. Table S2. Sequence information for the wild-type and mutated promoter regions of miR-124. Figure S1. Specific PCR products of miR-124 promoter region in mice. Figure S2. The original WB images.
