# Supplementary material for: Myostatin Orchestrates miR-124-Mediated Epigenetic Silencing of ITGB1 to Suppress Skeletal Muscle Growth
Source: Biomolecules. 2026 Jul 10;16(7):1012. doi: 10.3390/biom16071012 (PMC13406805; doi:10.3390/biom16071012)
Supplement: Supplementary file 1 [file biomolecules-16-01012-s001.zip › biomolecules-4395874-supplementary.pdf]

Table S1. Information of primers or oligonucleotides used in the present study (Species characteristics: *Mus musculus*)

Gene	primer sequences (5′-3′)	usage
miR-124	F: GAATGAGCGGTTCGAGAGTGTC	PCR
	R: CGGTGAAAATAAAGAACCTCGATG	
miR-124 mimics	UAAGGCACGCGGUGAAUGCC	
mimics NC	UUGUACUACACAAAAGUACU	
miR-124 inhibitor	GGCAUUCACCGCGUGCCUUA	
inhibitor NC	CAGUACUUUUGUGUAGUACA	
RPLP0	F: AGATTCGGGATATGCTGTTGGC	qRT-PCR
	R: TCGGGTCCTAGACCAGTGTTC	
ITGB1	F: ATGCCAAATCTTGCGGAGAAT	qRT-PCR
	R: TTTGCTGCGATTGGTGACATT	
MyoG	F: GCAGGCTCAAGAAAGTGAATGA	qRT-PCR
	R: TAGGCGCTCAATGTACTGGAT	
MyHC	F: GAGTTCATTGACTTCGGGATGG	qRT-PCR
	R: TGCTGCTCATACAGCTTGTTCTTG	
miR-124	GTCGTATCCAGTGCAGGGTCCGAGGTA	Reverse transcription
	TTCGCACTGGATACGACGGCATT	
gRNA1	GGTCGCACCCGGTCTAGGCAAGG	CRISPR editing
gRNA2	CGTCCGCGAGGTCGGCACTGCGG	CRISPR editing

Table S2. Sequence information for the wild-type and mutated promoter regions of miR-124 (Species characteristics: *Mus musculus*)

Item	Sequence information
**miR-124-3p-WT**	CCTCAGTTCTCAAGGACACCTCCGAGAGGAGGCGGCAGAGCCGGTGT CGGGTGACGTCACCGCGCGCCCCAGTGATAATCGCCCGGTGCCGGAGC CGAGCGCGGATACGAGCGGAGGCAGCGGCGGCGGCGGCGAGCGCGGG GACGGTCGCACCCGGTCTAGGCAAGGAGAGTGGGAGTCAGACTCCCG CAGTGCCGACCTCGCGGACGCCGGGCTCCCACCCGGCCCTCAGGAATT TGCCCGGCTTCCTTGGCACTGTCCCTGCGCTCTCCTCCACCCGGGGCCG TAGCGTGCGGGTCCTGCGGGCGCCAAGAAGGAGGCGGAGGCGGGATC GGGCGACAGCGCAGGCTGTGCTGCGGAGCGGAGGCGGCAACCGAGAA GCAGCAGGCGGCGGCGGCTCGCGCCCGGACGGGTAACTGCGCTGGCG GCCCGTGCCGGGGCGAGCCGGTCTCTCAGGTCACCCCGTCTTTCTCTC CTCGAGCAGCTTCTCGCCAGCCAGAGGCGTAAGAGAAGAGAGCTCGG CGGGTTGTAGAAAGAGACTGCTTTTCATCTCCGAACATCGAGGTTCTTT ATTTTCACCGCACTCACGCACTCCTGGTGG
**miR-124-3p-MUT**	CCTCAGTTCTCAAGGACACCTCCGAGAGGAGGCGGCAGAGCCGGTGT CGGGTGACGTCACCGCGCGCCCCAGTGATAATCGCCCGGTGCCGGAGC CGAGCGCGGATACGAGCGGAGGCAGCGGCGGCGGCGGCGAGCGCGGG GACGGTCGCACCCGGTCTAGGCAAGGAGAGTGGGAGTCAACTTCCCG CAGTGCCGACCTCGCGGACGCCGGGCTCCCACCCGGCCCTCAGGAATT TGCCCGGCTTCCTTGGCACTGTCCCTGCGCTCTCCTCCACCCGGGGCCG TAGCGTGCGGGTCCTGCGGGCGCCAAGAAGGAGGCGGAGGCGGGATC GGGCGACAGCGCAGGCTGTGCTGCGGAGCGGAGGCGGCAACCGAGAA GCAGCAGGCGGCGGCGGCTCGCGCCCGGACGGGTAACTGCGCTGGCG GCCCGTGCCGGGGCGAGCCGGTCTCTCAGGTCACCCCGTCTTTCTCTC CTCGAGCAGCTTCTCGCCAGCCAGAGGCGTAAGAGAAGAGAGCTCGG CGGGTTGTAGAAAGAGACTGCTTTTCATCTCCGAACATCGAGGTTCTTT ATTTTCACCGCACTCACGCACTCCTGGTGG

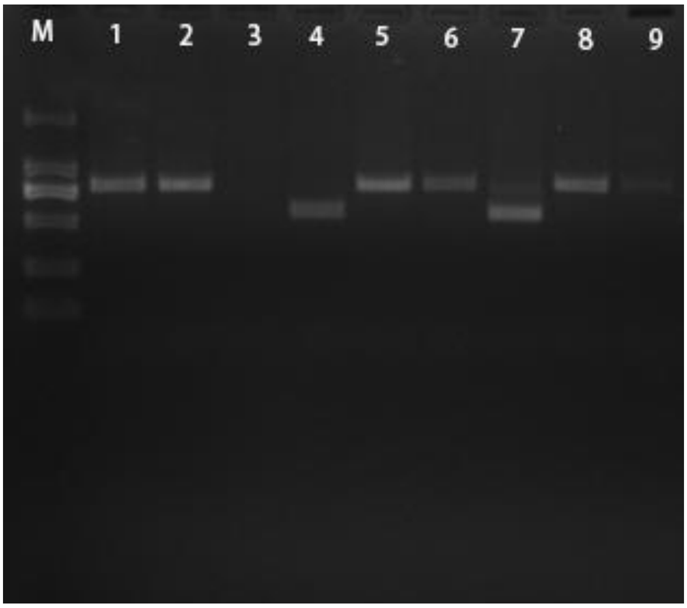

Figure S1. Specific PCR products of miR-124 promoter region in mice

Note: Lines 1, 2, 5, 6, 8, and 9: PCR products of wild-type mice; Line 4: PCR products of homozygous knockout mice; Line 7: PCR products of heterozygous knockout mice; M: DL2000 marker.

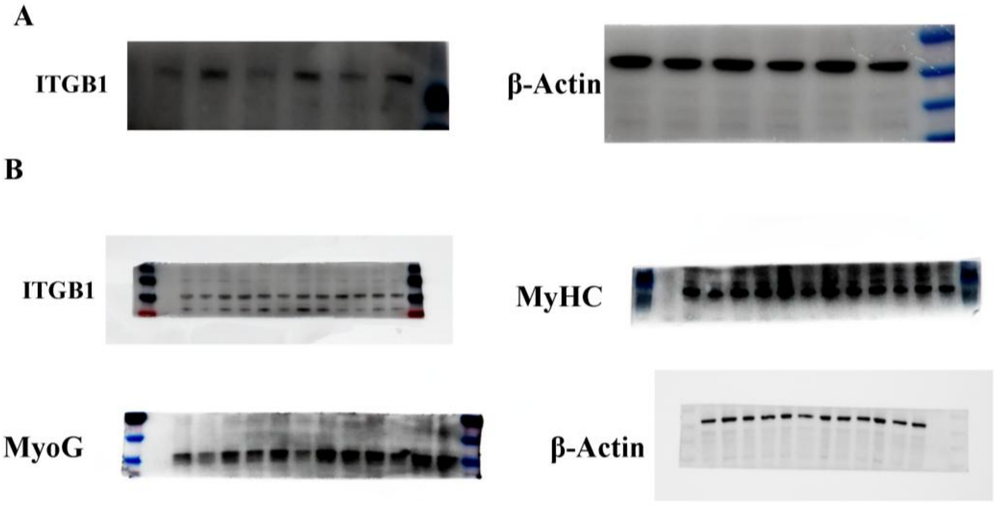

Figure S2. The original WB images

(A) The original WB images of Figure 1B;（B） The original WB images of Figure 5E;
